# Post-GWAS Functional Characterization of Susceptibility Variants for Chronic Lymphocytic Leukemia

**DOI:** 10.1371/journal.pone.0029632

**Published:** 2012-01-03

**Authors:** Fenna C. M. Sillé, Reuben Thomas, Martyn T. Smith, Lucia Conde, Christine F. Skibola

**Affiliations:** Division of Environmental Health Sciences, School of Public Health, University of California, Berkeley, California, United States of America; Dartmouth College, United States of America

## Abstract

Recent genome-wide association studies (GWAS) have identified several gene variants associated with sporadic chronic lymphocytic leukemia/small lymphocytic lymphoma (CLL/SLL). Many of these CLL/SLL susceptibility loci are located in non-coding or intergenic regions, posing a significant challenge to determine their potential functional relevance. Here, we review the literature of all CLL/SLL GWAS and validation studies, and apply eQTL analysis to identify putatively functional SNPs that affect gene expression that may be causal in the pathogenesis of CLL/SLL. We tested 12 independent risk loci for their potential to alter gene expression through *cis*-acting mechanisms, using publicly available gene expression profiles with matching genotype information. Sixteen SNPs were identified that are linked to differential expression of *SP140*, a putative tumor suppressor gene previously associated with CLL/SLL. Three additional SNPs were associated with differential expression of *DACT3* and *GNG8*, which are involved in the WNT/β-catenin- and G protein-coupled receptor signaling pathways, respectively, that have been previously implicated in CLL/SLL pathogenesis. Using *in silico* functional prediction tools, we found that 14 of the 19 significant eQTL SNPs lie in multiple putative regulatory elements, several of which have prior implications in CLL/SLL or other hematological malignancies. Although experimental validation is needed, our study shows that the use of existing GWAS data in combination with eQTL analysis and *in silico* methods represents a useful starting point to screen for putatively causal SNPs that may be involved in the etiology of CLL/SLL.

## Introduction

**Figure 1:**
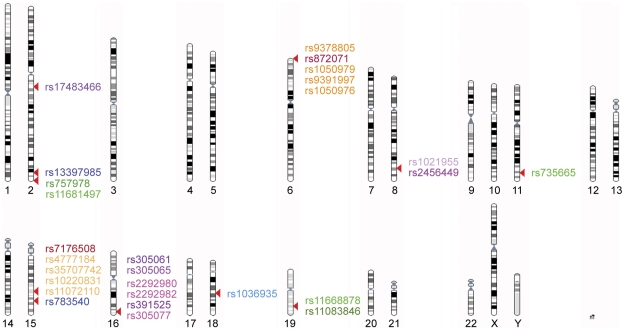
CLL/SLL susceptibility loci identified through genome-wide association studies. Karyotype depicts CLL/SLL-associated SNPs and SNPs in LD with those SNPs that were identified through previous genome-wide association studies (GWAS) and follow-up studies. Independent loci are color-coded with the primary GWAS SNP in dark and SNPs in LD in a lighter shade. Chromosome locations are based on chromosome build 37.1 GRCh37.

Chronic lymphocytic leukemia/small lymphocytic lymphoma (CLL/SLL) is the third most common type of non-Hodgkin lymphoma in Western countries [1], and exhibits one of the strongest familial aggregations of all malignancies [2]. Four genome-wide association studies (GWAS) [3], [4], [5], [6] and GWAS validation studies [7], [8], [9], [10], [11], [12] have identified several novel low-penetrance susceptibility alleles associated with sporadic and familial CLL/SLL. Together, these studies identified 12 independent loci in 11 novel susceptibility regions for CLL/SLL at 2q13, 2q37.1, 2q37.3, 6p25.3, 8q24.21, 11q24, 15q23, 15q25.2, 16q24.1, 18q21.1 and 19q13.32 [3], [4], [6], [7], [8], [9], [10], [11], [12]. With the exception of rs7169431 on 15q21.3, all original SNPs identified by the initial GWAS [4], [12] were validated either directly or indirectly through other SNPs in linkage disequilibrium (LD), in at least one of the other GWAS [3], [6] or in other independent, large-scale [7], [9], [11] or pooled [8] studies of Caucasian ancestry (Table 1 and Figure 1). Three of these SNPs, rs872071 (6p25.3, *IRF4*), rs13397985 (2q37.1, *SP140*) and rs17483466 (2q13, *ACOXL*), also were significantly associated with CLL/SLL in a Chinese population [10] . Similar to GWAS in other diseases, many of these CLL/SLL susceptibility loci are located in non-coding or intergenic regions with unknown biological relevance. This has prompted the need for a post-GWAS functional characterization of validated CLL/SLL risk alleles.

**Table 1:** CLL/SLL susceptibility loci identified through genome-wide association studies and follow-up studies.

Risk locus	SNP ID	r^2^	Chr Pos GRCh37 (bp)	Major/minor allele	Nearest gene(s)	SNP location	OR (risk allele)^a^	(95% CI) (risk allele)^a^	*P-*trend^a^	Ref.	Further validation ref.
2q13	**rs17483466**		111797458	A/**G**	*ACOXL, BCL2L11*	Intron 10 of *ACOXL*	1.39	(1.25–1.53)	2.36E-10	[4]	[6], [10], [11]
2q37.1	**rs13397985**		231091223	T**/G**	*SP140, SP110*	Intron 1 of *SP140*	1.41	(1.26–1.57)	5.40E-10	[4]	[6], [9], [10], [11]
2q37.3	**rs757978**		242371101	G/**A**	*FARP2*	Exon 9	1.39	(1.25–1.56)	2.11E-09	[12]	[6]
	*rs11681497* b	1	242344333	A/**G**	*FARP2*	Intron 4	1.38	(1.24–1.54)	4.53E-09	[12]	
6p25.3	**rs872071**		411064	A/**G**	*IRF4*	3′UTR	1.54	(1.41–1.69)	1.91E-20	[4]	[3], [6], [7], [9], [10], [11]
	*rs9378805* b	0.74	417727	A/**C**	*IRF4*	∼10 kb from 3′UTR	1.51	(1.38–1.65)	4.62E-19	[4]	[3], [6], [9], [11]
	*rs1050979* c	0.99	410417	A/**G**	*IRF4*	3′UTR	1.59	(1.43–1.78)	3.68E-17	[7]	
	*rs9391997* c	0.99	409119	A/**G**	*IRF4*	3′UTR	1.59	(1.42–1.77)	5.61E-17	[7]	
	*rs1050976* c	0.99	408079	C/**T**	*IRF4*	3′UTR	1.58	(1.41–1.76)	2.13E-16	[7]	
8q24.21	**rs2456449**		128192981	A/**G**	*–*	–	1.26	(1.17–1.35)	7.84E-10	[12]	[6]
	*rs2466024* b	0.75	128188019	G/**A**	*–*	–	1.2	(1.12–1.29)	4.60E-07	[12]	
11q24.1	**rs735665**		123361397	G/**A**	*GRAMD1B*	∼50 kb centromeric	1.45	(1.31–1.61)	3.78E-12	[4]	[3], [6], [9], [11]
15q23	**rs7176508**		70018990	G/**A**	*–*	–	1.37	(1.26–1.50)	4.54E-12	[4]	[6], [9], [11]
	*rs11072110*	>0.9	69987882	**T**/C	*–*	*–*	1.54	(1.24–1.92)	1.00E-04	[11]	
	*rs10220831*	>0.9	69992114	C/T/**G**	*–*	*–*	1.56	(1.25–1.95)	9.00E-05	[11]	
	*rs35707742*	>0.9	70004352	**G**/C	*–*	*–*	1.52	(1.22–1.90)	2.10E-04	[11]	
	*rs4777184*	>0.9	70007323	C/**T**	*–*	*–*	1.52	(1.22–1.89)	2.10E-04	[11]	
15q25.2	**rs783540** d		83254708	A/**G**	*CPEB1*	Intron 2	1.17	(1.11–1.24)	1.10E-07	[12]	[8]
16q24.1	**rs305061**		85975659	C/**T**	*IRF8*	∼19 kb telomeric	1.22	(1.12–1.32)	3.60E-07	[12]	[6]
	*rs305065*	0.93	85973866	**C**/G	*IRF8*	∼17 kb telomeric	0.77	(0.61–0.97)	2.37E-02	[6]	
	**rs391525**		85944439	A/**G**	*IRF8*	Intron 3	0.64	(0.55–0.74)	3.16E-09	[6]	
	*rs305077*	0.99	85943466	T/**C**	*IRF8*	Intron 3	0.66	(0.57–0.77)	3.37E-08	[6]	
	*rs2292980*	0.99	85945076	T/**C**	*IRF8*	Intron 3	0.66	(0.56–0.76)	1.89E-08	[6]	
	*rs2292982*	0.99	85944823	T/**G**	*IRF8*	Intron 3	0.64	(0.55–0.74)	3.16E-09	[6]	
18q21.1	**rs1036935** d		47843534	C**/T**	*CXXC1, MBD1*	Telomeric	1.16	(1.09–1.25)	1.30E-05	[12]	[8]
19q13.32	**rs11083846**		47207654	G/**A**	*PRKD2*, *STRN4*	Intron 3 of *PRKD2*	1.35	(1.22–1.49)	3.96E-09	[4]	[9]
	*rs11668878* b	0.27	47268373	G/**T**	*FKRP, SLC1A5*	Intergenic	1.37	(1.21–1.55)	3.65E-07	[4]	

*Notes:* In regular font are original independent SNPs identified through GWAS. In italics font are SNPs in LD with the original GWAS SNPs. In bold font are independently validated SNPs used for eQTL analysis and risk alleles as called by the primary study. Nearest gene(s) map within ∼200 kb of each SNP.

a OR, CI and *P*-trend quoted are per copy of risk allele (bold in column 5) from all data combined in the primary study. *P-*trend, significance of the association between each SNP and risk of CLL/SLL.

b Conditional analysis reportedly provided no evidence for an independent role compared to original SNP [4], [7], [12].

c Acquired after fine-scale mapping.

d Significance obtained from combined analysis from refs [12] and [8].

*Abbreviations:* LD, Linkage disequilibrium; OR, odds ratio; CLL/SLL, chronic lymphocytic leukemia/small lymphocytic lymphoma; CI, confidence interval.

Extensive inter-individual differences in gene expression exist in humans [13] that may account for an important fraction of phenotypic differences, including susceptibility to complex disorders such as CLL/SLL. Familial aggregation patterns in humans have unequivocally demonstrated an inherited contribution towards these phenotypic traits [13]. Loci responsible for this genetic control are known as expression quantitative trait loci (eQTL). The identification of eQTL is an emerging area in genomic studies, particularly with the integration of genome-wide SNP data and gene expression profiles. Several landmark eQTL studies in humans have been recently conducted that show that gene expression can be affected by polymorphisms in *cis*- or *trans*-regulatory regions [14] or by exonic variants that alter transcript stability or splicing [15]. Here, we reviewed the literature for all CLL/SLL GWAS and validation studies and applied eQTL analysis to identify putatively functional SNPs that affect gene expression and may be causal in the pathogenesis of CLL/SLL. We also conducted *in silico* functional analysis on these SNPs to explore the potential associated regulatory mechanisms that may be involved in CLL/SLL development.

## Results

### CLL/SLL eQTL analysis

**Figure 2:**
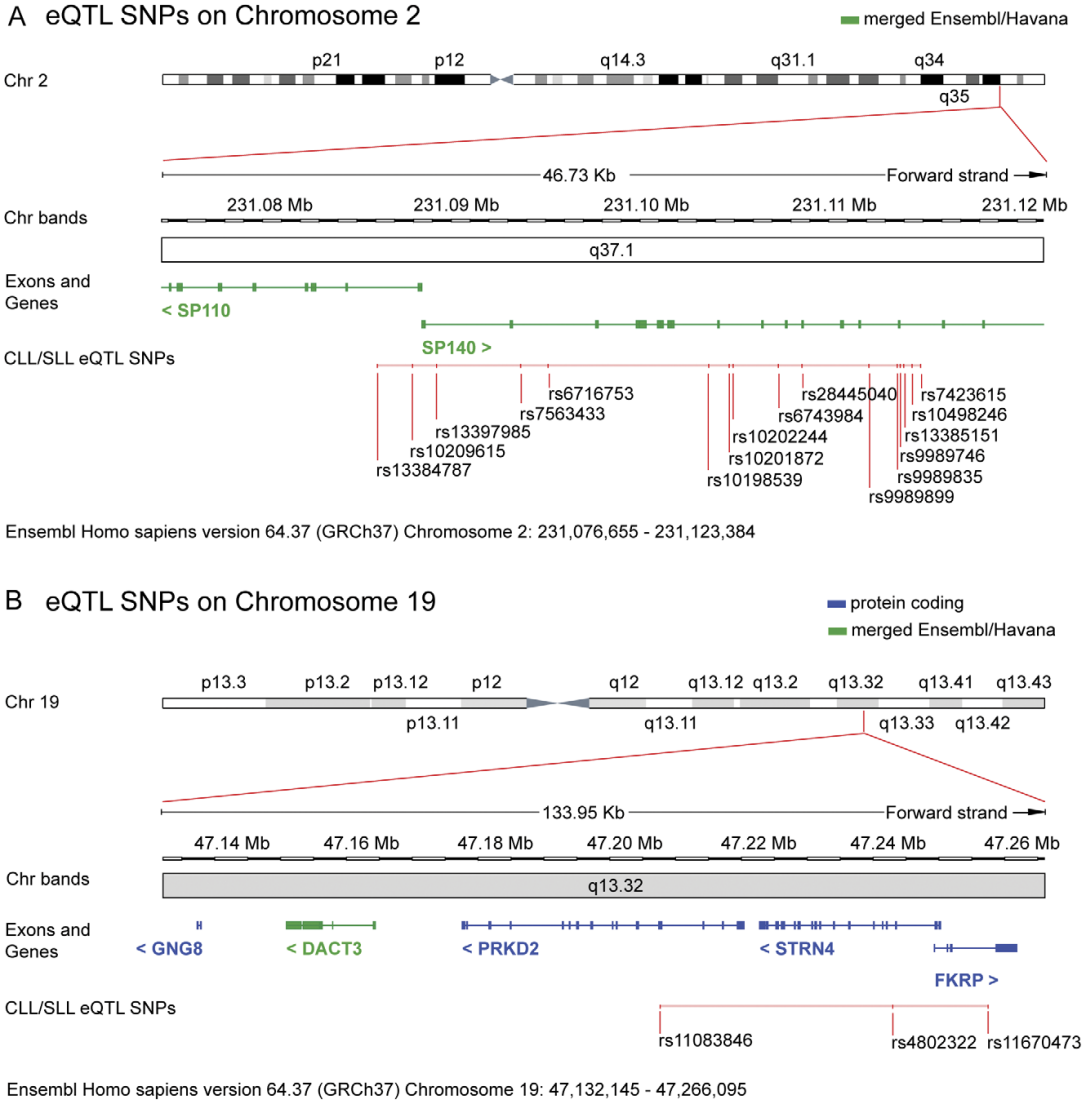
CLL/SLL-associated SNPs that significantly alter gene expression. Expression quantitative trait loci (eQTL) analysis identified 19 significant CLL/SLL-associated SNPs linked to differential expression of *SP140* on chromosome 2 (A), and *DACT3* and *GNG8* on chromosome 19 (BH<0.20) (B). eQTL SNPs are depicted on a partial chromosome map that includes the differentially expressed gene(s). Chromosome locations are based on chromosome build 37.1 GRCh37.

Using publicly available gene expression profile databases [16] with matching genotype information, we conducted an eQTL analysis on the 12 known GWAS CLL/SLL SNPs that were validated in independent studies (depicted in bold in Table 1) and included SNPs in LD (pairwise r^2^≥0.8) to test for their potential to alter gene expression through *cis*-acting mechanisms (Table S1). We identified two previously reported SNPs (rs13397985 and rs11083846) and 17 novel SNPs in two independent regions that were significantly associated with altered gene expression, assuming a threshold of 20% false positive ratio (BH<0.20) (Table 2 and Figure 2A and B). The minor alleles of 16 of these SNPs were linked to decreased *SP140* expression in the eQTL analysis. Two SNPs, rs11083846 and rs4802322, were significantly associated with a higher expression of two distinct genes, *DACT3* and *GNG8*, and rs11670473 was associated with increased *DACT3* expression.

**Table 2:** CLL/SLL-associated SNPs that alter gene expression through *cis*-acting mechanisms.

Differential gene expression	SNP ID	r^2^	Chr	Chr Pos GRCh37 (bp)	SNP location	Major/minor allele	Raw *P*-value	Adjusted *P*-value (BH)	Expression
*SP140*	rs6716753	0.95	2	231097129	Intron 1 of *SP140*	T/**C**	5.88E-03	0.186	↓
	rs6743984	0.95	2	231109329	Intron 5 of *SP140*	T/**C**	5.88E-03	0.186	↓
	rs7423615	1	2	231116874	Intron 10 of *SP140*	C/**T**	1.87E-03	0.114	↓
	rs7563433	1	2	231095678	Intron 1 of *SP140*	T/**C**	6.10E-03	0.186	↓
	rs9989746	0.89	2	231115790	Intron 10 of *SP140*	G/**A**	6.72E-04	0.082	↓
	rs9989835	1	2	231115630	Intron 9 of *SP140*	A/**T**	4.49E-03	0.171	↓
	rs9989899	1	2	231114131	Intron 9 of *SP140*	G/**A**	1.87E-03	0.114	↓
	rs10198539	0.89	2	231105646	Intron 5 of *SP140*	C/**T**	8.43E-04	0.086	↓
	rs10201872	0.89	2	231106724	Intron 5 of *SP140*	C/**T**	6.72E-04	0.082	↓
	rs10202244	0.89	2	231106935	Intron 5 of *SP140*	G/**T**	6.72E-04	0.082	↓
	rs10209615	0.83	2	231089943	Upstream of *SP140*	T/**C**	4.66E-04	0.082	↓
	rs10498246	1	2	231116417	Intron 10 of *SP140*	T/**A**	3.09E-03	0.157	↓
	rs13384787	0.89	2	231088084	Upstream of *SP140*	A/**C**	6.72E-04	0.082	↓
	rs13385151	1	2	231115976	Intron 10 of *SP140*	C/**T**	3.75E-03	0.164	↓
	**rs13397985**	-	2	231091223	Intron 1 of *SP140*	T/**G**	3.09E-03	0.157	↓
	rs28445040	1	2	231110582	Exon 7 of *SP140*	C/**T** c	1.87E-03	0.114	↓
*GNG8*	rs4802322^a^	0.95	19	47242992	Intron 1 of STRN4	**A**/G	6.63E-03	0.193	↑
	**rs11083846** b	-	19	47207654	Intron 5 of *PRKD2*	G/**A**	3.34E-03	0.157	↑
*DACT3*	rs4802322^a^	0.95	19	47242992	Intron 1 of *STRN4*	G/**A**	4.01E-03	0.164	↑
	**rs11083846** b	-	19	47207654	Intron 5 of *PRKD2*	G/**A**	1.85E-03	0.114	↑
	rs11670473	0.88	19	47257481	Intron 3 of *FKRP*	G/**A**	5.01E-03	0.180	↑

*Notes:* Depicted are CLL/SLL-associated SNPs and SNPs in LD that are significantly linked to differential gene expression (BH<0.20). Highlighted in bold are the original GWAS SNPs and the risk allele, or the minor allele when the risk allele is not known.

a,b Same SNP influencing expression of two distinct genes.

c The synonymous rs28445040 variation (TCC→TCT) does not lead to a substitution for the serine ([Ser]→[Ser]) at amino acid position 223.

*Abbreviations:* BH, Benjamini-Hochberg; ChrPos, chromosome position; CLL/SLL, chronic lymphocytic leukemia/small lymphocytic lymphoma.

### Screening for putative functionality of CLL/SLL eQTL SNPs

Using the *in silico* prediction tools F-SNP [17] and is-rSNP [18], we identified 41 putative regulatory elements for 12 of the 16 significant eQTL SNPs associated with altered *SP140* expression and for two of the three eQTL SNPs associated with *DACT3* or *GNG8* expression (Table S2). Because variations within evolutionarily conserved regions are more likely to be associated with gene expression phenotypes [19], [20], we also tested if the eQTL SNPs were located in genomic regions significantly conserved between *Homo sapiens*, *Mus musculus* or *Rattus norvegicus* (Table S2). Using the ECR browser [21], we found six SNPs in evolutionarily conserved regions (∼31.6% of our top eQTL SNPs), as expected based on prior predictions [19], [22]. Predictions for regulatory elements were found for three of these SNPs (rs13384787, rs9989746, and rs11670473, Table S2). To test if the functional predictions were likely to be biologically relevant, we searched the literature for prior evidence that the predicted regulatory elements were previously associated with any cancer, hematologic cancers and CLL/SLL, in particular (Table S2). Out of the 41 regulatory elements that may be influenced by the eQTL SNPs, more than half have previously been associated with hematologic cancers or CLL/SLL (see references in Table S2), supporting the biological plausibility of our findings.

## Discussion

Recent GWAS [3], [4], [5], [6] and validation studies [7], [8], [9], [10], [11], [12] have identified 12 independent loci associated with CLL/SLL risk. To further elucidate the functional importance of these and other CLL/SLL risk alleles, we conducted an eQTL analysis and applied *in silico* methods to explore the functional relevance of independently validated CLL/SLL GWAS SNPs and SNPs in high LD. Our analyses support the potential functionality of two previously reported SNPs and identified 17 novel putatively causal SNPs that either alter *SP140*, *DACT3* or *GNG8* gene expression. We also identified 41 putative regulatory elements that may be affected by these eQTL SNPs, many of which have been previously associated with hematologic malignancies or CLL/SLL.

### Factors predicted to alter SP140 expression in CLL/SLL

The *SP140* GWAS SNP, rs13397985, and 15 eQTL SNPs in LD were associated with significantly lower *SP140* expression. *SP140* is an appealing candidate for CLL/SLL susceptibility as it is restricted to lymphoid cells, specifically expressed in all mature B cells and plasma cell lines [23]. Similar to its homolog, SP100, SP140 contains a zinc-finger motif and a bromodomain suggesting that it plays a role in chromatin-mediated regulation of gene expression [23], [24]. Although it is currently unknown which genes are regulated by SP140, it is possible that SP140 regulates the expression of genes involved in CLL/SLL development. Homology with SP100 also suggests that SP140 may exert tumor suppressor activity [25]. This may provide a biologically feasible mechanism for the increased risk of CLL/SLL associated with reduced *SP140* expression. Moreover, as SP140 confers resistance to viruses [26], it may influence CLL/SLL risk through modulation of responses to antigenic stimulation.

Twelve of the 16 significant eQTL SNPs associated with altered *SP140* expression were located in potential regulatory elements. Two of these, rs13384787 and rs9989746, are in evolutionarily conserved regions. The most relevant findings for rs13384787 and rs9989746 are the disruption of a potential binding site for NIT2 and decreased binding potential for KLF4, respectively. Both transcription factors are putative tumor suppressor proteins [27], [28]; thus, a reduction in their binding potential might play a biologically relevant role in the pathogenesis of CLL/SLL.

Several eQTL SNPs in non-conserved regions are predicted to alter potential binding sites for regulatory elements that have been either directly or indirectly implicated in the pathogenesis of CLL/SLL and other hematological malignancies (See references in Table S2). Although experimental validation is needed, the tumor suppressors/pro-apoptotic factors, MZF1 [29] and the ubiquitous transcription factor STAT1 [30] may be biologically relevant as decreased binding interactions might lower *SP140* expression.

The microarray data used for the eQTL analysis is based on expression changes in lymphoblastoid cell lines. It is therefore possible that *in vitro* conditions may have affected gene expression levels. Thus, eQTL analyses will need to be followed up using gene expression data from fresh healthy lymphocytes once available. In addition, these lymphoblastoid cell lines were generated by Epstein-Barr virus transformation, which may theoretically contribute to the observed down-regulation of SP140, as SP140 is reportedly involved in mediating virus resistance [26]. Associations between CLL/SLL and several viruses, including Epstein-Barr virus have been suggested. However, no conclusive evidence of a causal relationship exists [31].

### Factors predicted to affect *DACT3* and *GNG8* expression in CLL/SLL

Three SNPs, rs4802322, rs11083846 and rs11670473 were associated with up-regulation of *DACT3*, a negative regulator of Wnt/β-catenin signaling that decreases activation of β-catenin-responsive genes [32]. Although activated Wnt/β-catenin signaling may suppress apoptosis in CLL/SLL cells [33], [34] and DACT3 inhibits this signaling pathway, our findings suggest that suppression of Wnt//β-catenin signaling in pre-CLL/SLL cells may increase susceptibility to CLL/SLL. We also found that rs4802322 and rs11083846 were associated with increased expression of *DACT3* and *GNG8*. Heterotrimeric G proteins such as GNG8 communicate extracellular signals received by G protein-coupled receptors to intracellular effector proteins (reviewed in [35]). GNG8 is involved in chemokine signaling that controls leukocyte transendothelial migration and plays a role in several pathways in the glutamatergic, cholinergic, GABAergic and dopaminergic synapses of the nervous system (KEGG) [36]. Interestingly, certain G Proteins and G protein-coupled receptor signaling pathways are associated with high relapse rate in CLL/SLL patients [37]. Moreover, they have been implicated in CLL/SLL cell migration [38] and may suppress apoptosis in CLL/SLL cells [39].

Of those SNPs localized to evolutionarily conserved regions, only rs11670473 is predicted to alter potential binding sites for several regulatory elements. Heat shock factors (HSF) and a putative “cap” binding site may be the most biologically relevant elements identified. HSF and heat shock proteins are known to play a role in tumorigenesis [40], [41]. Transcription [42] and mRNA translation [43] are initiated at the cap site. Interestingly, the cap-binding protein eIF4E and its repressor 4E-BP have been involved in numerous cancers [44] including CLL/SLL [45]. Although speculative, increased binding of HSF or cap-binding proteins and up-regulation of *DACT3* expression may decrease Wnt/β-catenin signaling and subsequently render cells susceptible to CLL/SLL transformation.

In this report we demonstrate that eQTL analysis of existing GWAS data, in combination with *in silico* functional predictions, is a powerful method to identify putative functional risk alleles and to explore potential causal mechanisms in the etiology of CLL/SLL. Our data highlight 19 SNPs that influence differential expression of *SP140* a putative tumor suppressor gene, *DACT3*, a negative regulator of the Wnt/β-catenin signaling pathway and *GNG8*, involved in G-protein coupled receptor and neurotransmitter signaling pathways. We identified 41 different regulatory elements that may be involved. Because an association with cancer can be made for almost any predicted regulatory element, we combined this method with a comparison of evolutionarily conserved regions to increase the likelihood of causality. Using this approach, we selectively identified two putative causal SNPs (rs13384787 and rs9989746) that influence *SP140* and one (rs11670473) that influences *DACT3* expression in association with CLL/SLL risk. Finally, we were able to identify several transcription factors whose function could theoretically be altered by the CLL/SLL risk alleles rs13384787, rs9989746 and rs11670473. Experimental studies will be needed to verify the functionality of the observed eQTL SNPs and to validate their biological relevance to help unravel the molecular mechanisms behind these potentially causal associations.

## Materials and Methods

### Study identification

A literature search of CLL/SLL GWAS and validation studies was conducted using the electronic database PubMed. We limited our search to entries on human studies from January 1992 up to the end of July 2011 (www.ncbi.nlm.nih.gov/pubmed). Our search strategy used the following keywords: chronic lymphocytic leukemia/small lymphocytic lymphoma (CLL/SLL) AND polymorphisms or single nucleotide polymorphisms (SNPs) AND genome-wide association (GWA). We searched for any additional studies in the bibliographies of identified publications, including review articles. A similar literature search was conducted to find studies reporting the involvement of an identified regulatory element with cancer, hematologic cancer or CLL/SLL. We used the following keywords in PubMed: “the name of the identified regulatory element” AND cancer or hematologic cancer or leukemia or chronic lymphocytic leukemia.

### CLL/SLL eQTL

For the eQTL analysis, one representative SNP per independently validated loci was selected (Table 1). The preprocessed and normalized microarray data [16] corresponding to each of the 60 unrelated European individuals from the HapMap project (Coriell, Camden, New Jersey, United States) [46] was acquired from the publicly accessible Gene Expression Omnibus (GEO) database [47] (http://www.ncbi.nlm.nih.gov/geo; accession number: GSE6536). SNPs linked to each representative SNP (*r* ^2^≥0.8) were identified using the European population (2009-04_rel27) linkage disequilibrium files downloaded from the HapMap project website (http://hapmap.ncbi.nlm.nih.gov). In total, 120 SNPs in the CLL/SLL-associated loci were then associated with the different genes that had probes on the microarray platform. Separate analyses to determine potential eQTL were carried out for each of the probes for a given gene. We limited our eQTL analysis to the detection of *cis*-regulatory regions, whereby the association between a SNP and a gene was made if it was located in the predefined chromosomal range between 300 kb upstream of the transcription start site of the corresponding gene and 100 kb downstream of the transcription end site of this gene. This resulted in a list of 613 SNP-gene pairs. The genotype for each of the unrelated European samples at each SNP location was obtained from the (2010-08_phaseII+III) genotype files on the HapMap project website (http://hapmap.ncbi.nlm.nih.gov). The access of information from the HapMap project files was done in Java [48]. The top candidates from our eQTL analysis are displayed in Table 2. Risk alleles for the eQTL SNPs found in LD with original GWAS SNPs were determined by matching genotype ratios with those from the original GWAS SNPs.

### Statistics

A linear regression between the number of minor alleles (0, 1, or 2) for a given SNP and the associated gene expression for each of the 60 samples was performed. The *P*-values obtained from testing the potential associations between the 613 SNP-gene pairs were subjected to multiple testing corrections using Benjamini-Hochberg (BH) False-Discovery Rate (FDR) procedure [49]. Associations were deemed significant at BH<0.20 [50], [51]. Linear regression was done using the *javastat* package [52] in Java. False Discovery Rate analysis was done using the multtest package [53] in the R statistical environment [54].

### Bioinformatics

We searched the Kyoto Encyclopedia of Genes and Genomes (KEGG) database (updated September 20, 2011) [36] for biological relevant pathways that include the differentially expressed genes identified in the eQTL analysis. The F-SNP database [17] (http://compbio.cs.queensu.ca/F-SNP/), which integrates 16 bioinformatics tools and databases, was used to predict functional effects on protein coding, splicing regulation, transcriptional regulation and post translation. Functional significance scores (FS) are defined by F-SNP, which ranges between 0 and 1. A FS of 0 means none of the tools predict a deleterious effect; whereas a FS of 1 suggests all tools predict a deleterious effect. FS were deemed significant if FS≥0.5, although it has to be taken into account that still 45% of disease-related SNPs were previously found to be <0.5 [55]. Therefore, SNPs with lower FS scores were still considered potentially functional when located in an evolutionarily conserved region (ECR) determined using the ECR browser (http://ecrbrowser.dcode.org) [21]. Since evolutionarily conserved regions are potentially more likely to indicate regions of functional importance, we performed a cross-species sequence comparison of the eQTL SNP target regions, using ECR browser. Regions overlapping the eQTL SNPs (SNP chromosome location +/−100 bp) in the human genome (*Homo sapiens*) were compared with mouse (*Mus musculus*) and rat (*Rattus norvegicus*). Alignments were considered significant when the evolutionarily conserved region met predefined length and identity criteria (100 nucleotides and at least 70% identity with either mouse or rat). As a cross-reference for the transcription factor results within F-SNP, we used is-rSNP [18] (http://www.genomics.csse.unimelb.edu.au/product-is-rSNP-service.php) to predict whether any of the eQTL SNPs would map to a potential transcription factor binding site. The is-rSNP prediction tool uses the non-redundant human TF database JASPAR [56] to first determine if any of the two SNP alleles are significantly predicted to be localized in a potential transcription factor binding site. This is determined based on binding scores computed using Position Weighted Matrices for the chosen transcription factor. For those potential transcription factors, is-rSNP calculates whether any of the two SNP alleles significantly alters the binding score. The is-rSNP tool uses a standard cut-off of *P*<0.05 for Benjamini-Hochberg [49] corrected *P*-values of the observed difference between the alleles.

### Karyotype

A karyotype displaying the reported CLL/SLL risk loci was generated using Ensembl release 64 - September 2011, Karyotype build CRCh37 (http://uswest.ensembl.org/Homo_sapiens/Location/Genome). Similarly, Ensembl release 64 - September 2011, Karyotype build CRCh37 was used to generate position overviews of significant eQTL SNPs on chromosome 2 and 19. SNP identifiers were manually added in Adobe Illustrator CS3.

## Supporting Information

Table S1 **CLL/SLL-associated SNPs in relation to gene expression.** *Notes:* Depicted are CLL/SLL-associated SNPs and SNPs in LD (r^2^≥0.8), which are linked to gene expression. Highlighted in bold are the CLL/SLL-associated SNPs and SNPs in LD, which are significantly linked to differential gene expression (BH<0.20). *Abbreviations:* BH, Benjamini-Hochberg; Chr Pos, chromosome position.
(XLSX)

Table S2 **Potential** ***cis*** **-acting regulatory elements affected by CLL/SLL-associated SNPs identified by expression quantitative trait loci analysis.** *Notes:* Depicted are CLL/SLL-associated SNPs and SNPs in LD that are significantly linked to differential gene expression (BH<0.20). Highlighted in bold are the SNPs located in evolutionarily conserved regions and the risk allele or the minor allele when the risk allele is not known. ^a^ Predicted change in binding score for putative regulatory element relative to the minor allele. ^b^ Difference *P*-value: significance of the change in binding score between the two SNP alleles, calculated by the is-rSNP tool. ^c^ Adjusted difference *P*-value (BH): The Benjamini-Hochberg corrected *P*-value of the observed change in binding score between the two SNP alleles, calculated by the is-rSNP tool (shown are elements with BH-corrected *P*<0.05). ^d^ Functional significance score calculated by F-SNP tool. ^e^ Evolutionarily conserved region based on 100 nucleotides with at least 70% identity, determined using the ECR browser. ^f^ If present, prior evidence that the predicted *cis*-acting regulatory element plays a role in carcinogenesis. ^g^ If present, prior evidence that the predicted *cis*-acting regulatory element is associated with hematologic malignancies. ^h^ If present, prior evidence that the predicted *cis*-acting regulatory element is associated with CLL/SLL. *Abbreviations:* BH, Benjamini-Hochberg; CLL/SLL, chronic lymphocytic leukemia/small lymphocytic lymphoma; CP, cap site; ECR, evolutionarily conserved region; ESR, exonic splicing regulator; FC, frame shift coding; FS, functional significance; OG, oncogenic; POG, proto-oncogenic; SS, splicing site; TF, transcription factor; TS, tumor suppressive.
(DOCX)
