# Supplementary material for: Post-GWAS Functional Characterization of Susceptibility Variants for Chronic Lymphocytic Leukemia
Source: PLoS One. 2012 Jan 3;7(1):e29632. doi: 10.1371/journal.pone.0029632 (PMC3250464; doi:10.1371/journal.pone.0029632)
Supplement: Table S2 — Potential cis -acting regulatory elements affected by CLL/SLL-associated SNPs identified by expression quantitative trait loci analysis. Notes: Depicted are CLL/SLL-associated SNPs and SNPs in LD that are significantly linked to differential gene expression (BH<0.20). Highlighted in bold are the SNPs located in evolutionarily conserved regions and the risk allele or the minor allele when the risk allele is not known. a Predicted change in binding score for putative regulatory element relative to the minor allele. b Difference P-value: significance of the change in binding score between the two SNP alleles, calculated by the is-rSNP tool. c Adjusted difference P-value (BH): The Benjamini-Hochberg corrected P-value of the observed change in binding score between the two SNP alleles, calculated by the is-rSNP tool (shown are elements with BH-corrected P<0.05). d Functional significance score calculated by F-SNP tool. e Evolutionarily conserved region based on 100 nucleotides with at least 70% identity, determined using the ECR browser. f If present, prior evidence that the predicted cis-acting regulatory element plays a role in carcinogenesis. g If present, prior evidence that the predicted cis-acting regulatory element is associated with hematologic malignancies. h If present, prior evidence that the predicted cis-acting regulatory element is associated with CLL/SLL. Abbreviations: BH, Benjamini-Hochberg; CLL/SLL, chronic lymphocytic leukemia/small lymphocytic lymphoma; CP, cap site; ECR, evolutionarily conserved region; ESR, exonic splicing regulator; FC, frame shift coding; FS, functional significance; OG, oncogenic; POG, proto-oncogenic; SS, splicing site; TF, transcription factor; TS, tumor suppressive. (DOCX) [file pone.0029632.s002.docx]

**Table S2. Potential *cis*-acting regulatory elements affected by CLL/SLL-associated SNPs identified by expression quantitative trait loci analysis.**

| **SNP ID** | **Diff. gene expression** | **Allele (**A/**a)** | **Regulatory element** | **Type** | **∆ binding ^a^** | **Difference *P*-value ^b^** | **Adjusted difference *P*-value (BH) ^c^** | **FS score ^d^** | **Role in carcinogenesis ^f^, ref.** | **Prior association with hematologic malignancy ^g^, ref.** | **Prior (indirect) association with CLL/SLL ^h^, ref.** |
| --- | --- | --- | --- | --- | --- | --- | --- | --- | --- | --- | --- |
| rs6716753 | *SP140* | T/**C** | - | - | - | - | - | - | - | - | - |
| rs6743984 | *SP140* | T/**C** | Hand1::E2A | TF | ↑ | 4.18E-05 | 2.26E-03 | - | OG / TS, [4,5,6,7,8,9] | ALL / Lymphoma, [1] | Indirect through E2A: anti-apoptotic and regulates oncogene CD38 in CLL, [2,3] |
|  |  |  | Pou5f1 | TF | ↓ | 1.14E-04 | 6.15E-03 | - | OG, proliferative, metastatic, [11,12,13,14,15] | Myeloblastic leukemia, [10] | - |
|  |  |  | SOX2 | TF | ↓ | 2.83E-05 | 1.53E-03 | - | OG, anti-apoptotic, [16,17,18,19,20] | - | - |
| rs7423615 ^e^ | *SP140* | C/**T** | - | - | - | - | - | - | - | - | - |
| rs7563433 | *SP140* | T/**C** | FOXA1 | TF | ↑ | 1.73E-04 | 9.36E-03 | - | OG, [21,22,23,24,25,26] | - | - |
| rs9989746  ^e^ | *SP140* | G/**A**/**T** | EWSR1-FLI1 | TF | ↓ | 3.78E-05 | 2.04E-03 | - | OG, [27,28,29] | - | - |
|  |  |  | KLF4 | TF | ↓ | 2.35E-04 | 1.27E-02 | - | OG / TS, [31] / [32,33] | BCR-ABL ALL, [30] | - |
|  |  |  | NF-kappaB | TF | ↓ | 3.10E-05 | 1.68E-03 | - | OG, anti-apoptotic, Reviewed in [34] | Multiple hematologic cancers, Reviewed in [34] | Regulates anti-apoptotic genes in CLL, [35,36,37,38,39,40] |
|  |  |  | NFKB1 | TF | ↓ | 5.97E-05 | 3.22E-03 | - | OG, anti-apoptotic, [38,41,42] | - | Regulates anti-apoptotic genes in CLL, [38,41,42] |
|  |  |  | REL | TF | ↓ | 3.94E-05 | 2.13E-03 | - | OG, anti-apoptotic, [38,41,42] | - | Regulates anti-apoptotic genes in CLL, [38,41,42] |
|  |  |  | RELA | TF | ↓ | 4.58E-05 | 2.47E-03 | - | OG, anti-apoptotic, [43,44,45] | - | Regulates anti-apoptotic genes in CLL, [43,44,45] |
| rs9989835 | *SP140* | A/**T** | NKX3-1 | TF | ↓ | 9.68E-05 | 5.23E-03 | - | TS, inhibits cell proliferation and invasion / OG, proliferative, [47,48,49] | T-ALL , [46]. | - |
|  |  |  | Nr2e3 | TF | ↓ | 3.39E-05 | 1.83E-03 | - | - | - | - |
| rs9989899 | *SP140* | G/**A** | PLAG1 | TF | ↓ | 1.75E-04 | 9.47E-03 | - | OG, anti-apoptotic, [53,54,55,56,57,58,59] | AML, [50]. | Epigenetic silencing of miRNA: overexpression of PLAG1, [51,52] |
| rs10198539 | *SP140* | C/**T** | - | - | - | - | - | - | - | - | - |
| rs10201872 | *SP140* | C/**T** | EBF1 | TF | ↓ | 3.29E-04 | 1.78E-02 | - | regulates TS and OG, [60] | ALL, [60] | - |
|  |  |  | TLX1::NFIC | TF | ↑ | 4.49E-05 | 2.42E-03 | - | OG, [61,62] | T-ALL, [61,62] | - |
| rs10202244 | *SP140* | G/**T** | MZF1_5-13 | TF | ↓ | 5.86E-05 | 3.16E-03 | - | TS, [64] | - | Indirect through LDOC1: apoptosis and poor survival of CLL patients, [63] |
|  |  |  | Spz1 | TF | ↓ | 4.37E-05 | 2.36E-03 | - | POG, [66] | - | Indirect through PCNA: short survival of CLL patients, [65] |
|  |  |  | TLX1::NFIC | TF | ↑ | 3.82E-05 | 2.06E-03 | - | OG, [61,62] | T-ALL, [61,62] | - |
| rs10209615 | *SP140* | T/**C** | HSF | TF | ↓ | - | - | 0.5 | OG, [72] | - | Heat shock proteins: Zap-70 degradation, apoptosis, impaired signaling in CLL cells, [67,68,69,70,71] |
|  |  |  | SOX2 | TF | ↓ | 1.66E-04 | 8.95E-03 | - | OG, anti-apoptotic, [16,17,18,19,20] | - | - |
|  |  |  | Sp1 | TF | Gain | - | - | 0.5 | OG, telomerase activation, sequestered by c-Myc causes repression of p21, [73,74,75] | Myeloid leukemia cells, [73,74,75] | - |
|  |  |  | TEAD1 | TF | ↓ | 3.02E-04 | 1.63E-02 | - | Biomarker, [76] | - | - |
| rs10498246 ^e^ | *SP140* | T/**A** | - | - | - | - | - | - | - | - | - |
| rs13384787 ^e^ | *SP140* | A/**C** | GATA-2 | TF | Gain | - | - | 0.5 | OG, [77,78] | ALL, CML, [77,78] | - |
|  |  |  | GATA-3 | TF | Gain | - | - | 0.5 | OG, [79] | - | - |
|  |  |  | Ik-2 | TF | Gain | - | - | 0.5 | Regulates FB1-E2A: OG, [80] | ALL, [80] | Indirect through E2A: anti-apoptotic and regulates oncogene CD38 in CLL, [2,3] |
|  |  |  | NIT2 | TF | Loss | - | - | 0.5 | TS, [81] | - | - |
| rs13385151 | *SP140* | C/**T** | ELK4 | TF | ↓ | 4.86E-05 | 2.62E-03 | - | OG, [82,83] | - | - |
|  |  |  | GABPA | TF | ↓ | 6.37E-04 | 3.44E-02 | - | Regulates pro-apoptotic TMS1/ASC: OG when epigenetically silenced, [84,85,86] | - | - |
|  |  |  | STAT1 | TF | ↓ | 2.62E-04 | 1.41E-02 | - | TS, apoptotic, inhibits cell differentiation and migration / OG, anti-apoptotic, Reviewed in [97] | Lymphoblastoid cells, AML, Burkitt's lymphoma, [87] | TS: apoptotic, inhibits CLL cell differentiation and migration / OG: anti-apoptotic with VGEFR, [88,89,90,91,92,93,94] / [95,96]. |
| **rs13397985** | *SP140* | T/**G** | FOXA1 | TF | ↓ | 1.89E-04 | 1.02E-02 | - | OG, [21,22,23,24,25,26] | - | - |
|  |  |  | FOXA2 | TF | ↓ | 6.50E-05 | 3.51E-03 | - | OG / TS, anti-metastatic, [98,99] / [23,100,101,102] | - | - |
|  |  |  | FOXQ1 | TF | ↓ | 1.97E-04 | 1.06E-02 | - | OG, proliferative, metastatic, [103,104,105,106] | - | - |
|  |  |  | SRF | TF | ↑ | 8.77E-04 | 4.74E-02 | - | OG, proliferative, anti-apoptotic, metastatic / TS, [108,109,110,111,112] | 5q- myelodysplastic syndromes, [107] | - |
| rs28445040 | *SP140* | C/**T** | hnRNP/PTB | ESR | Gain | - | - | 0.33 | OG, [114,115,116] | Multiple myeloma , [113] | - |
|  |  |  | NFIL3 | TF | ↑ | 3.98E-04 | 2.15E-02 | - | OG: regulated by E2A-HLF, [117] | ALL, [117] | - |
|  |  |  | SC35 | ESR | Loss | - | - | 0.33 | TS: silences cancer-associated genes through splicing, - | Erythroleukemia, [118] | Epigenetic silencing of E-cadherin in CLL, [119] |
|  |  |  | SC35 | ESR | ↓ | - | - | 0.33 | Idem | Idem | Idem, Idem |
| rs4802322 ^e^ | *DACT3, GNG8* | G/**A** | - | - | - | - | - | - | - | - | - |
| **rs11083846** | *DACT3, GNG8* | G/**A** | EWSR1-FLI1 | TF | ↓ | 1.05E-04 | 5.64E-03 | - | OG, [27,28,29] | - | - |
|  |  |  | RUNX1 | TF | ↓ | 2.20E-06 | 1.19E-04 | - | OG, [122,123] | AML and ALL, [120,121] | - |
|  |  |  | - | FC | - | - | - | 1 | - | - | - |
|  |  |  | - | SS | - | - | - | 1 | - | - | - |
| rs11670473 ^e^ | *DACT3* | G/**A** | Cap site | CS | ↑ | - | - | 0.208 | mRNA cap-binding protein eIF4E: OG, cell survival, anti-apoptotic, [126] | - | mRNA cap-binding protein eIF4E: OG, cell survival, anti-apoptotic, [124,125] |
|  |  |  | CdxA | TF | Gain | - | - | 0.208 | - | - | - |
|  |  |  | FOXC1 | TF | - | - | - | 0.208 | OG mutations / TS, [127] / [128] / [129] | - | - |
|  |  |  | HSF | TF | Gain | - | - | 0.208 | OG, [72] | - | Heat shock proteins: Zap-70 degradation, apoptosis, impaired signaling in CLL cells, [67,68,69,70,71] |
|  |  |  | T | TF | ↓ | 1.32E-04 | 7.13E-03 | - | OG, progression to metastasis, [130] | - | - |

Notes: Depicted are CLL/SLL-associated SNPs and SNPs in LD that are significantly linked to differential gene expression (BH < 0.20). Highlighted in bold are the SNPs located in evolutionarily conserved regions and the risk allele or the minor allele when the risk allele is not known. ^a^ Predicted change in binding score for putative regulatory element relative to the minor allele. ^b^ Difference *P*-value: significance of the change in binding score between the two SNP alleles, calculated by the is-rSNP tool. ^c^ Adjusted difference *P*-value (BH): The Benjamini-Hochberg corrected *P*-value of the observed change in binding score between the two SNP alleles, calculated by the is-rSNP tool (shown are elements with BH-corrected *P* < 0.05). ^d^ Functional significance score calculated by F-SNP tool. ^e^ Evolutionarily conserved region based on 100 nucleotides with at least 70% identity, determined using the ECR browser. ^f^ If present, prior evidence that the predicted *cis*-acting regulatory element plays a role in carcinogenesis. ^g^ If present, prior evidence that the predicted *cis*-acting regulatory element is associated with hematologic malignancies. ^h^ If present, prior evidence that the predicted *cis*-acting regulatory element is associated with CLL/SLL.

Abbreviations: BH, Benjamini-Hochberg; CLL/SLL, chronic lymphocytic leukemia / small lymphocytic lymphoma; CP, cap site; ECR, evolutionarily conserved region; ESR, exonic splicing regulator; FC, frame shift coding; FS, functional significance; OG, oncogenic; POG, proto-oncogenic; SS, splicing site; TF, transcription factor; TS, tumor suppressive.

**References**

1. Steininger A, Mobs M, Ullmann R, Kochert K, Kreher S, et al. (2011) Genomic loss of the putative tumor suppressor gene E2A in human lymphoma. J Exp Med 208: 1585-1593.

2. Kardava L, Yang Q, St Leger A, Foon KA, Lentzsch S, et al. (2011) The B lineage transcription factor E2A regulates apoptosis in chronic lymphocytic leukemia (CLL) cells. Int Immunol 23: 375-384.

3. Saborit-Villarroya I, Vaisitti T, Rossi D, D'Arena G, Gaidano G, et al. (2011) E2A is a transcriptional regulator of CD38 expression in chronic lymphocytic leukemia. Leukemia 25: 479-488.

4. Shin CM, Kim N, Jung Y, Park JH, Kang GH, et al. (2010) Role of Helicobacter pylori infection in aberrant DNA methylation along multistep gastric carcinogenesis. Cancer Sci 101: 1337-1346.

5. Yagi K, Akagi K, Hayashi H, Nagae G, Tsuji S, et al. (2010) Three DNA methylation epigenotypes in human colorectal cancer. Clin Cancer Res 16: 21-33.

6. Tellez CS, Shen L, Estecio MR, Jelinek J, Gershenwald JE, et al. (2009) CpG island methylation profiling in human melanoma cell lines. Melanoma Res 19: 146-155.

7. Imura M, Yamashita S, Cai LY, Furuta J, Wakabayashi M, et al. (2006) Methylation and expression analysis of 15 genes and three normally-methylated genes in 13 Ovarian cancer cell lines. Cancer Lett 241: 213-220.

8. Hagihara A, Miyamoto K, Furuta J, Hiraoka N, Wakazono K, et al. (2004) Identification of 27 5' CpG islands aberrantly methylated and 13 genes silenced in human pancreatic cancers. Oncogene 23: 8705-8710.

9. Martinez Hoyos J, Ferraro A, Sacchetti S, Keller S, De Martino I, et al. (2009) HAND1 gene expression is negatively regulated by the High Mobility Group A1 proteins and is drastically reduced in human thyroid carcinomas. Oncogene 28: 876-885.

10. Bunaciu RP, Yen A (2011) Activation of the aryl hydrocarbon receptor AhR Promotes retinoic acid-induced differentiation of myeloblastic leukemia cells by restricting expression of the stem cell transcription factor Oct4. Cancer Res 71: 2371-2380.

11. Linn DE, Yang X, Sun F, Xie Y, Chen H, et al. (2010) A Role for OCT4 in Tumor Initiation of Drug-Resistant Prostate Cancer Cells. Genes Cancer 1: 908-916.

12. Fujino T, Nomura K, Ishikawa Y, Makino H, Umezawa A, et al. (2010) Function of EWS-POU5F1 in sarcomagenesis and tumor cell maintenance. Am J Pathol 176: 1973-1982.

13. Du Z, Jia D, Liu S, Wang F, Li G, et al. (2009) Oct4 is expressed in human gliomas and promotes colony formation in glioma cells. Glia 57: 724-733.

14. Chang CC, Shieh GS, Wu P, Lin CC, Shiau AL, et al. (2008) Oct-3/4 expression reflects tumor progression and regulates motility of bladder cancer cells. Cancer Res 68: 6281-6291.

15. Zhu Z, Wen J, Zheng X, Wang D, Wang Q, et al. (2009) Expression of transcription factor Oct4 in bladder cancer cell line T24 and its effects on the biological characteristics of the cells. J Huazhong Univ Sci Technolog Med Sci 29: 73-76.

16. Fang X, Yoon JG, Li L, Yu W, Shao J, et al. (2011) The SOX2 response program in glioblastoma multiforme: an integrated ChIP-seq, expression microarray, and microRNA analysis. BMC Genomics 12: 11.

17. Jia X, Li X, Xu Y, Zhang S, Mou W, et al. (2011) SOX2 promotes tumorigenesis and increases the anti-apoptotic property of human prostate cancer cell. J Mol Cell Biol.

18. Li XL, Eishi Y, Bai YQ, Sakai H, Akiyama Y, et al. (2004) Expression of the SRY-related HMG box protein SOX2 in human gastric carcinoma. Int J Oncol 24: 257-263.

19. Rodriguez-Pinilla SM, Sarrio D, Moreno-Bueno G, Rodriguez-Gil Y, Martinez MA, et al. (2007) Sox2: a possible driver of the basal-like phenotype in sporadic breast cancer. Mod Pathol 20: 474-481.

20. Sholl LM, Long KB, Hornick JL (2010) Sox2 expression in pulmonary non-small cell and neuroendocrine carcinomas. Appl Immunohistochem Mol Morphol 18: 55-61.

21. Bernardo GM, Lozada KL, Miedler JD, Harburg G, Hewitt SC, et al. (2010) FOXA1 is an essential determinant of ERalpha expression and mammary ductal morphogenesis. Development 137: 2045-2054.

22. Mehta RJ, Jain RK, Leung S, Choo J, Nielsen T, et al. (2011) FOXA1 is an independent prognostic marker for ER-positive breast cancer. Breast Cancer Res Treat.

23. Song Y, Washington MK, Crawford HC (2010) Loss of FOXA1/2 is essential for the epithelial-to-mesenchymal transition in pancreatic cancer. Cancer Res 70: 2115-2125.

24. Zhang Y, Ali TZ, Zhou H, D'Souza DR, Lu Y, et al. (2010) ErbB3 binding protein 1 represses metastasis-promoting gene anterior gradient protein 2 in prostate cancer. Cancer Res 70: 240-248.

25. Sano M, Aoyagi K, Takahashi H, Kawamura T, Mabuchi T, et al. (2010) Forkhead box A1 transcriptional pathway in KRT7-expressing esophageal squamous cell carcinomas with extensive lymph node metastasis. Int J Oncol 36: 321-330.

26. Nucera C, Eeckhoute J, Finn S, Carroll JS, Ligon AH, et al. (2009) FOXA1 is a potential oncogene in anaplastic thyroid carcinoma. Clin Cancer Res 15: 3680-3689.

27. Ohno T, Rao VN, Reddy ES (1993) EWS/Fli-1 chimeric protein is a transcriptional activator. Cancer Res 53: 5859-5863.

28. May WA, Lessnick SL, Braun BS, Klemsz M, Lewis BC, et al. (1993) The Ewing's sarcoma EWS/FLI-1 fusion gene encodes a more potent transcriptional activator and is a more powerful transforming gene than FLI-1. Mol Cell Biol 13: 7393-7398.

29. France KA, Anderson JL, Park A, Denny CT (2011) Oncogenic Fusion Protein EWS/FLI1 Down-regulates Gene Expression by Both Transcriptional and Posttranscriptional Mechanisms. J Biol Chem 286: 22750-22757.

30. Kharas MG, Yusuf I, Scarfone VM, Yang VW, Segre JA, et al. (2007) KLF4 suppresses transformation of pre-B cells by ABL oncogenes. Blood 109: 747-755.

31. Rowland BD, Bernards R, Peeper DS (2005) The KLF4 tumour suppressor is a transcriptional repressor of p53 that acts as a context-dependent oncogene. Nat Cell Biol 7: 1074-1082.

32. Gao J, Sai N, Wang C, Sheng X, Shao Q, et al. (2011) Overexpression of chromokinesin KIF4 inhibits proliferation of human gastric carcinoma cells both in vitro and in vivo. Tumour Biol 32: 53-61.

33. Zhao W, Hisamuddin IM, Nandan MO, Babbin BA, Lamb NE, et al. (2004) Identification of Kruppel-like factor 4 as a potential tumor suppressor gene in colorectal cancer. Oncogene 23: 395-402.

34. Chaturvedi MM, Sung B, Yadav VR, Kannappan R, Aggarwal BB (2011) NF-kappaB addiction and its role in cancer: 'one size does not fit all'. Oncogene 30: 1615-1630.

35. Mihalcik SA, Tschumper RC, Jelinek DF (2010) Transcriptional and post-transcriptional mechanisms of BAFF-receptor dysregulation in human B lineage malignancies. Cell Cycle 9: 4884-4892.

36. Buggins AG, Pepper C, Patten PE, Hewamana S, Gohil S, et al. (2010) Interaction with vascular endothelium enhances survival in primary chronic lymphocytic leukemia cells via NF-kappaB activation and de novo gene transcription. Cancer Res 70: 7523-7533.

37. Watanabe N, Iwamura T, Shinoda T, Fujita T (1997) Regulation of NFKB1 proteins by the candidate oncoprotein BCL-3: generation of NF-kappaB homodimers from the cytoplasmic pool of p50-p105 and nuclear translocation. EMBO J 16: 3609-3620.

38. Endo T, Nishio M, Enzler T, Cottam HB, Fukuda T, et al. (2007) BAFF and APRIL support chronic lymphocytic leukemia B-cell survival through activation of the canonical NF-kappaB pathway. Blood 109: 703-710.

39. Liu Z, Hazan-Halevy I, Harris DM, Li P, Ferrajoli A, et al. (2011) STAT-3 activates NF-kappaB in chronic lymphocytic leukemia cells. Mol Cancer Res 9: 507-515.

40. Longo PG, Laurenti L, Gobessi S, Sica S, Leone G, et al. (2008) The Akt/Mcl-1 pathway plays a prominent role in mediating antiapoptotic signals downstream of the B-cell receptor in chronic lymphocytic leukemia B cells. Blood 111: 846-855.

41. Furman RR, Asgary Z, Mascarenhas JO, Liou HC, Schattner EJ (2000) Modulation of NF-kappa B activity and apoptosis in chronic lymphocytic leukemia B cells. J Immunol 164: 2200-2206.

42. Pickering BM, de Mel S, Lee M, Howell M, Habens F, et al. (2007) Pharmacological inhibitors of NF-kappaB accelerate apoptosis in chronic lymphocytic leukaemia cells. Oncogene 26: 1166-1177.

43. Hewamana S, Alghazal S, Lin TT, Clement M, Jenkins C, et al. (2008) The NF-kappaB subunit Rel A is associated with in vitro survival and clinical disease progression in chronic lymphocytic leukemia and represents a promising therapeutic target. Blood 111: 4681-4689.

44. Hewamana S, Lin TT, Rowntree C, Karunanithi K, Pratt G, et al. (2009) Rel a is an independent biomarker of clinical outcome in chronic lymphocytic leukemia. J Clin Oncol 27: 763-769.

45. Ougolkov AV, Bone ND, Fernandez-Zapico ME, Kay NE, Billadeau DD (2007) Inhibition of glycogen synthase kinase-3 activity leads to epigenetic silencing of nuclear factor kappaB target genes and induction of apoptosis in chronic lymphocytic leukemia B cells. Blood 110: 735-742.

46. Kusy S, Gerby B, Goardon N, Gault N, Ferri F, et al. (2010) NKX3.1 is a direct TAL1 target gene that mediates proliferation of TAL1-expressing human T cell acute lymphoblastic leukemia. J Exp Med 207: 2141-2156.

47. Gurel B, Ali TZ, Montgomery EA, Begum S, Hicks J, et al. (2010) NKX3.1 as a marker of prostatic origin in metastatic tumors. Am J Surg Pathol 34: 1097-1105.

48. Wang P, Ma Q, Luo J, Liu B, Tan F, et al. (2009) Nkx3.1 and p27(KIP1) cooperate in proliferation inhibition and apoptosis induction in human androgen-independent prostate cancer cells. Cancer Invest 27: 369-375.

49. Nagel S, Venturini L, Przybylski GK, Grabarczyk P, Schneider B, et al. (2011) Activation of Paired-homeobox gene PITX1 by del(5)(q31) in T-cell acute lymphoblastic leukemia. Leuk Lymphoma 52: 1348-1359.

50. Landrette SF, Kuo YH, Hensen K, Barjesteh van Waalwijk van Doorn-Khosrovani S, Perrat PN, et al. (2005) Plag1 and Plagl2 are oncogenes that induce acute myeloid leukemia in cooperation with Cbfb-MYH11. Blood 105: 2900-2907.

51. Pallasch CP, Patz M, Park YJ, Hagist S, Eggle D, et al. (2009) miRNA deregulation by epigenetic silencing disrupts suppression of the oncogene PLAG1 in chronic lymphocytic leukemia. Blood 114: 3255-3264.

52. Patz M, Pallasch CP, Wendtner CM (2010) Critical role of microRNAs in chronic lymphocytic leukemia: overexpression of the oncogene PLAG1 by deregulated miRNAs. Leuk Lymphoma 51: 1379-1381.

53. Astrom A, D'Amore ES, Sainati L, Panarello C, Morerio C, et al. (2000) Evidence of involvement of the PLAG1 gene in lipoblastomas. Int J Oncol 16: 1107-1110.

54. Gisselsson D, Hibbard MK, Dal Cin P, Sciot R, Hsi BL, et al. (2001) PLAG1 alterations in lipoblastoma: involvement in varied mesenchymal cell types and evidence for alternative oncogenic mechanisms. Am J Pathol 159: 955-962.

55. Ropke A, Kalinski T, Kluba U, von Falkenhausen U, Wieacker PF, et al. (2007) PLAG1 activation in lipoblastoma coinciding with low-level amplification of a derivative chromosome 8 with a deletion del(8)(q13q21.2). Cytogenet Genome Res 119: 33-38.

56. Brandal P, Bjerkehagen B, Heim S (2006) Rearrangement of chromosomal region 8q11-13 in lipomatous tumours: correlation with lipoblastoma morphology. J Pathol 208: 388-394.

57. Zatkova A, Rouillard JM, Hartmann W, Lamb BJ, Kuick R, et al. (2004) Amplification and overexpression of the IGF2 regulator PLAG1 in hepatoblastoma. Genes Chromosomes Cancer 39: 126-137.

58. Kandasamy J, Smith A, Diaz S, Rose B, O'Brien C (2007) Heterogeneity of PLAG1 gene rearrangements in pleomorphic adenoma. Cancer Genet Cytogenet 177: 1-5.

59. Matsuyama T, Grossman A, Mittrücker HW, Siderovski DP, Kiefer F, et al. (1995) Molecular cloning of LSIRF, a lymphoid-specific member of the interferon regulatory factor family that binds the interferon-stimulated response element (ISRE). Nucleic Acids Research 23: 2127-2136.

60. Heltemes-Harris LM, Willette MJ, Ramsey LB, Qiu YH, Neeley ES, et al. (2011) Ebf1 or Pax5 haploinsufficiency synergizes with STAT5 activation to initiate acute lymphoblastic leukemia. J Exp Med 208: 1135-1149.

61. De Keersmaecker K, Ferrando AA (2011) TLX1 induced T-cell acute lymphoblastic leukemia. Clin Cancer Res.

62. Ferrando AA, Neuberg DS, Dodge RK, Paietta E, Larson RA, et al. (2004) Prognostic importance of TLX1 (HOX11) oncogene expression in adults with T-cell acute lymphoblastic leukaemia. Lancet 363: 535-536.

63. Duzkale H, Schweighofer CD, Coombes KR, Barron LL, Ferrajoli A, et al. (2011) LDOC1 mRNA is differentially expressed in chronic lymphocytic leukemia and predicts overall survival in untreated patients. Blood 117: 4076-4084.

64. Gaboli M (2001) Mzf1 controls cell proliferation and tumorigenesis. Genes & Development 15: 1625-1630.

65. Faderl S, Keating MJ, Do K-A, Liang S-Y, Kantarjian HM, et al. (2002) Expression profile of 11 proteins and their prognostic significance in patients with chronic lymphocytic leukemia (CLL). Leukemia 16: 1045-1052.

66. Hsu S-H (2005) bHLH-zip Transcription Factor Spz1 Mediates Mitogen-Activated Protein Kinase Cell Proliferation, Transformation, and Tumorigenesis. Cancer Research 65: 4041-4050.

67. O'Hayre M, Salanga CL, Kipps TJ, Messmer D, Dorrestein PC, et al. (2010) Elucidating the CXCL12/CXCR4 signaling network in chronic lymphocytic leukemia through phosphoproteomics analysis. PLoS ONE 5: e11716.

68. Dempsey NC, Leoni F, Ireland HE, Hoyle C, Williams JH (2010) Differential heat shock protein localization in chronic lymphocytic leukemia. J Leukoc Biol 87: 467-476.

69. Valbuena JR, Rassidakis GZ, Lin P, Atwell C, Georgakis GV, et al. (2005) Expression of heat-shock protein-90 in non-Hodgkin's lymphomas. Mod Pathol 18: 1343-1349.

70. Vokes EE, Golomb HM, Samuels BL, Brownstein BH (1989) Heat shock proteins in normal and leukemic blood cells. J Interferon Res 9: 195-204.

71. Castro JE, Prada CE, Loria O, Kamal A, Chen L, et al. (2005) ZAP-70 is a novel conditional heat shock protein 90 (Hsp90) client: inhibition of Hsp90 leads to ZAP-70 degradation, apoptosis, and impaired signaling in chronic lymphocytic leukemia. Blood 106: 2506-2512.

72. Dai C, Whitesell L, Rogers AB, Lindquist S (2007) Heat shock factor 1 is a powerful multifaceted modifier of carcinogenesis. Cell 130: 1005-1018.

73. Liu L, Ishihara K, Ichimura T, Fujita N, Hino S, et al. (2009) MCAF1/AM is involved in Sp1-mediated maintenance of cancer-associated telomerase activity. J Biol Chem 284: 5165-5174.

74. Gartel AL, Ye X, Goufman E, Shianov P, Hay N, et al. (2001) Myc represses the p21(WAF1/CIP1) promoter and interacts with Sp1/Sp3. Proc Natl Acad Sci U S A 98: 4510-4515.

75. Munoz-Alonso MJ, Ceballos L, Bretones G, Frade P, Leon J, et al. (2011) MYC accelerates p21(CIP) -induced megakaryocytic differentiation involving early mitosis arrest in leukemia cells. J Cell Physiol.

76. Knight JF, Shepherd CJ, Rizzo S, Brewer D, Jhavar S, et al. (2008) TEAD1 and c-Cbl are novel prostate basal cell markers that correlate with poor clinical outcome in prostate cancer. Br J Cancer 99: 1849-1858.

77. Wu XL, Li YQ, Wang Z, Yang LJ, Chen SH, et al. (2005) [Expressions of transcription factor GATA-1 and GATA-2 genes in bone marrow stromal cells from patients with leukemia]. Zhongguo Shi Yan Xue Ye Xue Za Zhi 13: 70-75.

78. Zhang SJ, Shi JY, Li JY (2009) GATA-2 L359 V mutation is exclusively associated with CML progression but not other hematological malignancies and GATA-2 P250A is a novel single nucleotide polymorphism. Leuk Res 33: 1141-1143.

79. Ali A, Christie PT, Grigorieva IV, Harding B, Van Esch H, et al. (2007) Functional characterization of GATA3 mutations causing the hypoparathyroidism-deafness-renal (HDR) dysplasia syndrome: insight into mechanisms of DNA binding by the GATA3 transcription factor. Hum Mol Genet 16: 265-275.

80. Brambillasca F, Mosna G, Ballabio E, Biondi A, Boulukos KE, et al. (2001) Promoter analysis of TFPT (FB1), a molecular partner of TCF3 (E2A) in childhood acute lymphoblastic leukemia. Biochem Biophys Res Commun 288: 1250-1257.

81. Lin CH, Chung MY, Chen WB, Chien CH (2007) Growth inhibitory effect of the human NIT2 gene and its allelic imbalance in cancers. FEBS J 274: 2946-2956.

82. Makkonen H, Jaaskelainen T, Pitkanen-Arsiola T, Rytinki M, Waltering KK, et al. (2008) Identification of ETS-like transcription factor 4 as a novel androgen receptor target in prostate cancer cells. Oncogene 27: 4865-4876.

83. Miyagi Y, Sasaki T, Fujinami K, Sano J, Senga Y, et al. (2010) ETS family-associated gene fusions in Japanese prostate cancer: analysis of 194 radical prostatectomy samples. Mod Pathol 23: 1492-1498.

84. Atlas E, Stramwasser M, Whiskin K, Mueller CR (2000) GA-binding protein alpha/beta is a critical regulator of the BRCA1 promoter. Oncogene 19: 1933-1940.

85. Eichner LJ, Perry MC, Dufour CR, Bertos N, Park M, et al. (2010) miR-378( *) mediates metabolic shift in breast cancer cells via the PGC-1beta/ERRgamma transcriptional pathway. Cell Metab 12: 352-361.

86. Lucas ME, Crider KS, Powell DR, Kapoor-Vazirani P, Vertino PM (2009) Methylation-sensitive regulation of TMS1/ASC by the Ets factor, GA-binding protein-alpha. J Biol Chem 284: 14698-14709.

87. Weber-Nordt RM, Egen C, Wehinger J, Ludwig W, Gouilleux-Gruart V, et al. (1996) Constitutive activation of STAT proteins in primary lymphoid and myeloid leukemia cells and in Epstein-Barr virus (EBV)-related lymphoma cell lines. Blood 88: 809-816.

88. de Totero D, Meazza R, Zupo S, Cutrona G, Matis S, et al. (2006) Interleukin-21 receptor (IL-21R) is up-regulated by CD40 triggering and mediates proapoptotic signals in chronic lymphocytic leukemia B cells. Blood 107: 3708-3715.

89. Gowda A, Roda J, Hussain SR, Ramanunni A, Joshi T, et al. (2008) IL-21 mediates apoptosis through up-regulation of the BH3 family member BIM and enhances both direct and antibody-dependent cellular cytotoxicity in primary chronic lymphocytic leukemia cells in vitro. Blood 111: 4723-4730.

90. de Totero D, Meazza R, Capaia M, Fabbi M, Azzarone B, et al. (2008) The opposite effects of IL-15 and IL-21 on CLL B cells correlate with differential activation of the JAK/STAT and ERK1/2 pathways. Blood 111: 517-524.

91. Liang X, Moseman EA, Farrar MA, Bachanova V, Weisdorf DJ, et al. (2010) Toll-like receptor 9 signaling by CpG-B oligodeoxynucleotides induces an apoptotic pathway in human chronic lymphocytic leukemia B cells. Blood 115: 5041-5052.

92. Tomic J, Lichty B, Spaner DE (2011) Aberrant interferon-signaling is associated with aggressive chronic lymphocytic leukemia. Blood 117: 2668-2680.

93. Ugarte-Berzal E, Redondo-Munoz J, Eroles P, Del Cerro MH, Garcia-Marco JA, et al. (2010) VEGF/VEGFR2 interaction down-regulates matrix metalloproteinase-9 via STAT1 activation and inhibits B chronic lymphocytic leukemia cell migration. Blood 115: 846-849.

94. Battle TE, Frank DA (2003) STAT1 mediates differentiation of chronic lymphocytic leukemia cells in response to Bryostatin 1. Blood 102: 3016-3024.

95. Frank DA, Mahajan S, Ritz J (1997) B lymphocytes from patients with chronic lymphocytic leukemia contain signal transducer and activator of transcription (STAT) 1 and STAT3 constitutively phosphorylated on serine residues. J Clin Invest 100: 3140-3148.

96. Lee YK, Shanafelt TD, Bone ND, Strege AK, Jelinek DF, et al. (2005) VEGF receptors on chronic lymphocytic leukemia (CLL) B cells interact with STAT 1 and 3: implication for apoptosis resistance. Leukemia 19: 513-523.

97. Cheon H, Yang J, Stark GR (2011) The functions of signal transducers and activators of transcriptions 1 and 3 as cytokine-inducible proteins. J Interferon Cytokine Res 31: 33-40.

98. Lehner F, Kulik U, Klempnauer J, Borlak J (2010) Inhibition of the liver enriched protein FOXA2 recovers HNF6 activity in human colon carcinoma and liver hepatoma cells. PLoS ONE 5: e13344.

99. Qi J, Nakayama K, Cardiff RD, Borowsky AD, Kaul K, et al. (2010) Siah2-dependent concerted activity of HIF and FoxA2 regulates formation of neuroendocrine phenotype and neuroendocrine prostate tumors. Cancer Cell 18: 23-38.

100. Tang Y, Shu G, Yuan X, Jing N, Song J (2011) FOXA2 functions as a suppressor of tumor metastasis by inhibition of epithelial-to-mesenchymal transition in human lung cancers. Cell Res 21: 316-326.

101. Yu KS, Jo JY, Kim SJ, Lee Y, Bae JH, et al. (2011) Epigenetic regulation of the transcription factor Foxa2 directs differential elafin expression in melanocytes and melanoma cells. Biochem Biophys Res Commun 408: 160-166.

102. Akagi T, Luong QT, Gui D, Said J, Selektar J, et al. (2008) Induction of sodium iodide symporter gene and molecular characterisation of HNF3 beta/FoxA2, TTF-1 and C/EBP beta in thyroid carcinoma cells. Br J Cancer 99: 781-788.

103. Bieller A, Pasche B, Frank S, Glaser B, Kunz J, et al. (2001) Isolation and characterization of the human forkhead gene FOXQ1. DNA Cell Biol 20: 555-561.

104. Kaneda H, Arao T, Tanaka K, Tamura D, Aomatsu K, et al. (2010) FOXQ1 is overexpressed in colorectal cancer and enhances tumorigenicity and tumor growth. Cancer Res 70: 2053-2063.

105. Zhang H, Meng F, Liu G, Zhang B, Zhu J, et al. (2011) Forkhead transcription factor foxq1 promotes epithelial-mesenchymal transition and breast cancer metastasis. Cancer Res 71: 1292-1301.

106. Qiao Y, Jiang X, Lee ST, Karuturi RK, Hooi SC, et al. (2011) FOXQ1 regulates epithelial-mesenchymal transition in human cancers. Cancer Res 71: 3076-3086.

107. Eisenmann KM, Dykema KJ, Matheson SF, Kent NF, DeWard AD, et al. (2009) 5q- myelodysplastic syndromes: chromosome 5q genes direct a tumor-suppression network sensing actin dynamics. Oncogene 28: 3429-3441.

108. Kwon CY, Kim KR, Choi HN, Chung MJ, Noh SJ, et al. (2010) The role of serum response factor in hepatocellular carcinoma: implications for disease progression. Int J Oncol 37: 837-844.

109. Yu W, Feng S, Dakhova O, Creighton CJ, Cai Y, et al. (2011) FGFR-4 Arg388 Enhances Prostate Cancer Progression via Extracellular Signal-Related Kinase and Serum Response Factor Signaling. Clin Cancer Res 17: 4355-4366.

110. Hu Q, Guo C, Li Y, Aronow BJ, Zhang J (2011) LMO7 Mediates Cell-Specific Activation of the Rho-Myocardin-Related Transcription Factor-Serum Response Factor Pathway and Plays an Important Role in Breast Cancer Cell Migration. Mol Cell Biol 31: 3223-3240.

111. Choi HN, Kim KR, Lee JH, Park HS, Jang KY, et al. (2009) Serum response factor enhances liver metastasis of colorectal carcinoma via alteration of the E-cadherin/beta-catenin complex. Oncol Rep 21: 57-63.

112. Kim HJ, Kim KR, Park HS, Jang KY, Chung MJ, et al. (2009) The expression and role of serum response factor in papillary carcinoma of the thyroid. Int J Oncol 35: 49-55.

113. Cobbold LC, Wilson LA, Sawicka K, King HA, Kondrashov AV, et al. (2010) Upregulated c-myc expression in multiple myeloma by internal ribosome entry results from increased interactions with and expression of PTB-1 and YB-1. Oncogene 29: 2884-2891.

114. He X, Ee PL, Coon JS, Beck WT (2004) Alternative splicing of the multidrug resistance protein 1/ATP binding cassette transporter subfamily gene in ovarian cancer creates functional splice variants and is associated with increased expression of the splicing factors PTB and SRp20. Clin Cancer Res 10: 4652-4660.

115. Jin W, Bruno IG, Xie TX, Sanger LJ, Cote GJ (2003) Polypyrimidine tract-binding protein down-regulates fibroblast growth factor receptor 1 alpha-exon inclusion. Cancer Res 63: 6154-6157.

116. David CJ, Chen M, Assanah M, Canoll P, Manley JL (2010) HnRNP proteins controlled by c-Myc deregulate pyruvate kinase mRNA splicing in cancer. Nature 463: 364-368.

117. Yeung J, O'Sullivan E, Hubank M, Brady HJ (2004) E4BP4 expression is regulated by the t(17;19)-associated oncoprotein E2A-HLF in pro-B cells. Br J Haematol 125: 560-567.

118. Shi J, Hu Z, Pabon K, Scotto KW (2008) Caffeine regulates alternative splicing in a subset of cancer-associated genes: a role for SC35. Mol Cell Biol 28: 883-895.

119. Sharma S, Lichtenstein A (2009) Aberrant splicing of the E-cadherin transcript is a novel mechanism of gene silencing in chronic lymphocytic leukemia cells. Blood 114: 4179-4185.

120. Ter Elst A, Ma B, Scherpen FJ, de Jonge HJ, Douwes J, et al. (2011) Repression of vascular endothelial growth factor expression by the runt-related transcription factor 1 in acute myeloid leukemia. Cancer Res 71: 2761-2771.

121. Gaidzik VI, Bullinger L, Schlenk RF, Zimmermann AS, Rock J, et al. (2011) RUNX1 mutations in acute myeloid leukemia: results from a comprehensive genetic and clinical analysis from the AML study group. J Clin Oncol 29: 1364-1372.

122. Inoue KI, Ito Y (2011) Neuroblastoma cell proliferation is sensitive to changes in levels of RUNX1 and RUNX3 protein. Gene.

123. Edfeldt K, Bjorklund P, Akerstrom G, Westin G, Hellman P, et al. (2011) Different gene expression profiles in metastasizing midgut carcinoid tumors. Endocr Relat Cancer 18: 479-489.

124. Saad H, Belle R, Morales J, Cosson B, Mulner-Lorillon O, et al. (2007) [Initiation factors eIF4: from sea urchin embryonic development to chronic lymphocytic leukemia]. J Soc Biol 201: 307-315.

125. Kodali D, Rawal A, Ninan MJ, Patel MR, Mesa H, et al. (2011) Expression and Phosphorylation of Eukaryotic Translation Initiation Factor 4E Binding Protein 1 in B-Cell Lymphomas and Reactive Lymphoid Tissues. Arch Pathol Lab Med 135: 365-371.

126. Holland EC, Sonenberg N, Pandolfi PP, Thomas G (2004) Signaling control of mRNA translation in cancer pathogenesis. Oncogene 23: 3138-3144.

127. Chakrabarti S, Kaur K, Rao KN, Mandal AK, Kaur I, et al. (2009) The transcription factor gene FOXC1 exhibits a limited role in primary congenital glaucoma. Invest Ophthalmol Vis Sci 50: 75-83.

128. Zhou Y, Kato H, Asanoma K, Kondo H, Arima T, et al. (2002) Identification of FOXC1 as a TGF-beta1 responsive gene and its involvement in negative regulation of cell growth. Genomics 80: 465-472.

129. Ray PS, Wang J, Qu Y, Sim MS, Shamonki J, et al. (2010) FOXC1 is a potential prognostic biomarker with functional significance in basal-like breast cancer. Cancer Res 70: 3870-3876.

130. Palena C, Polev DE, Tsang KY, Fernando RI, Litzinger M, et al. (2007) The human T-box mesodermal transcription factor Brachyury is a candidate target for T-cell-mediated cancer immunotherapy. Clin Cancer Res 13: 2471-2478.
